# Systematic Review of Medicinal Plants Used for Treatment of Diabetes in Human Clinical Trials: An ASEAN Perspective

**DOI:** 10.1155/2021/5570939

**Published:** 2021-10-13

**Authors:** Nazurah Hamizah Salleh, Ihsan Nazurah Zulkipli, Hartini Mohd Yasin, Fairuzeta Ja'afar, Norhayati Ahmad, Wan Amir Nizam Wan Ahmad, Siti Rohaiza Ahmad

**Affiliations:** ^1^PAPRSB Institute of Health Sciences, Universiti Brunei Darussalam, Jalan Tungku Link, Gadong BE1410, Brunei Darussalam; ^2^Chemical Sciences, Faculty of Science, Universiti Brunei Darussalam, Jalan Tungku Link, Gadong BE1410, Brunei Darussalam; ^3^Environmental and Life Sciences, Faculty of Science, Universiti Brunei Darussalam, Jalan Tungku Link, Gadong BE1410, Brunei Darussalam; ^4^Institute for Biodiversity and Environmental Research, Universiti Brunei Darussalam, Jalan Tungku Link, Gadong BE1410, Brunei Darussalam; ^5^School of Health Sciences, University Sains Malaysia, Gelugor, Malaysia

## Abstract

Traditionally, there are some medicinal plants believed to treat diabetes, as they have been proven in research studies to possess antidiabetic properties, such as improved insulin sensitivity and hypoglycemic activities, due to their high level of phenolic compounds, flavonoids, terpenoids, alkaloids, and glycosides. We conducted a systematic review to identify potential medicinal plants used during human clinical trials in the Association of Southeast Asian Nation (ASEAN) countries on prediabetic or type 2 diabetic individuals and to potentially identify any bioactive compounds involved in effectively treating symptoms of diabetes such as lowering of blood glucose. A total of 1209 reference titles were retrieved from four selected databases (Science Direct, Scopus, Springer Link, and PubMed) and only three met the inclusion criteria. Upon evaluation of the selected articles, four medicinal plants were identified: turmeric (*Curcuma longa*), garlic (*Allium sativum* L.), bitter melon (*Momordica charantia*), and Rosella flower (*Hibiscus sabdariffa* L.). Of these, only the bitter melon study did not show any significant change in the blood glucose of participants after intervention. This review demonstrates the limitations in published articles of human clinical trials for medicinal plants' intervention for diabetes. Upon further investigations on the four identified medicinal plants included in the animal studies, the findings showed positive effects in the management of diabetes, such as hyperglycemia. Hence, further testing and standardization of the methods in the studies can be suggested for human clinical trials for reliable data collections such as methods of extract preparation, duration of intervention, and conditions set for the study design.

## 1. Introduction

Diabetes mellitus (DM) is increasingly prevalent and continues to be a leading health burden worldwide, with consistent increases in mortality due to the various complications associated with the disease, such as coronary heart disease, nephropathy, and neurodegeneration. Although diagnosis and management of the disease have improved considerably; according to the World Health Organization (WHO), the lack of resources, specialized services, and skilled health workers in Southeast Asian countries contribute to the steady rise of the diabetes epidemic, especially type 2 DM in adults [1]. Hence, there is a need to develop low-cost solutions for diabetes, especially in underserved areas in different regions [1].

With increasing global expenditure on diabetes of over USD 700 billion (10%) [2] and the adverse effects of prolonged consumption of conventional drugs [3], natural and less invasive products are gaining popularity for prevention and as therapeutic measures. Some natural products have been shown to effectively control the abnormality in carbohydrate metabolism, further reducing the deterioration of overall health of affected individuals; have also shown to be relatively low in cost; and have minimal to no side effects at all [4, 5].

Traditionally, it is known plants naturally contain healing properties for various ailments, have been used for generations, and play an important role in leading modern medicine to where it is now. Generations worth of knowledge provide invaluable insights into understanding the various uses of different medicinal plants in different cultures around the world and have assisted researchers in identifying potencies of these plants to cure diseases [6–8].

By the early nineteenth century, centuries of passed down knowledge on the traditional uses of plants and the methods of herbal preparation were better understood as apothecaries were able to isolate bioactive compounds, mainly alkaloids such as quinine, caffeine, and cocaine from their natural sources, which gradually have shifted to synthetic development of these phytochemicals for better efficiency and potency [9, 10], whereby, for the latter, some known drugs are aspirin from willow bark, steroids from Mexican yam, and opium from poppy [9, 11]. Although the cost is lowered as opposed to isolating compounds from natural sources, over time they have shown side effects that could potentially harm consumers [7].

There are some medicinal plants believed to treat diabetes, and scientific studies have reported certain medicinal plants do contain antidiabetic properties, such as improved insulin sensitivity and hypoglycemic activities [12]. This is often associated with their high level of phenolic compounds, flavonoids, terpenoids, alkaloids, and glycosides, which can improve insulin secretions as well as control blood glucose [13]. Atanasov et al. [9] reported that some of the compounds known to have beneficial effects on carbohydrate metabolism are quercetin and resveratrol, which are found in onion (*Allium cepa* L.) and grapevine *(Vitis vinifera* L.), respectively.

In 2017, the Association of Southeast Asian Nation (ASEAN) secretariat published an updated compilation of available medicinal plants for the 10 ASEAN countries, except for Singapore and Vietnam, which reported various health benefits of these plants such as treatment for inflammation, hemorrhoids, and diabetes [14]. Although the plants' chemical constituents, medical uses, and traditional practices were mentioned, most remain unsupported, or clinical evidence is still limited or unavailable to support this information and health claims.

The preclinical studies that were already conducted on some of these plants were notably done mostly on animals rather than on humans. Therefore, this study will attempt to investigate existing human clinical studies conducted in Southeast Asian countries, to identify medicinal plants that have proven to be beneficial for the management of type 2 diabetes mellitus, particularly in lowering blood glucose.

The study objective is to conduct a systematic review of published research conducted on medicinal plants in human studies in ASEAN countries on prediabetic or type 2 diabetic patients and to eventually identify bioactive compounds of these medicinal plants that contribute to the effectiveness in treating diabetes.

## 2. Material and Methods

### 2.1. Literature Research

To assess findings on clinical evidence in different ASEAN countries on the usage of medicinal plants that have shown some form of antidiabetic effects in human studies, an extensive literature search has been conducted and completed using multiple databases (Science Direct, Scopus, Springer Link, and PubMed) covering all published journals from the 1900s to the most current.

Key search items used in this study included, “diabet^*∗∗*^ OR “type 2 diabetes mellitus” OR “pre-diabet^*∗∗*^”, “ASEAN OR Brunei OR Indonesia OR Laos OR Malaysia OR Philippines OR Thailand OR Vietnam OR Myanmar OR Singapore OR Cambodia”, “medicinal plant” OR “medicinal herb” OR “herbal”.

We have limited our search to focus on medicinal plants in ASEAN countries and human clinical studies, whereby only original research papers with completed clinical trials and assessment are considered. Mendeley 1.19.4 was used as a database manager.

### 2.2. Study Inclusion

**Figure 1:**
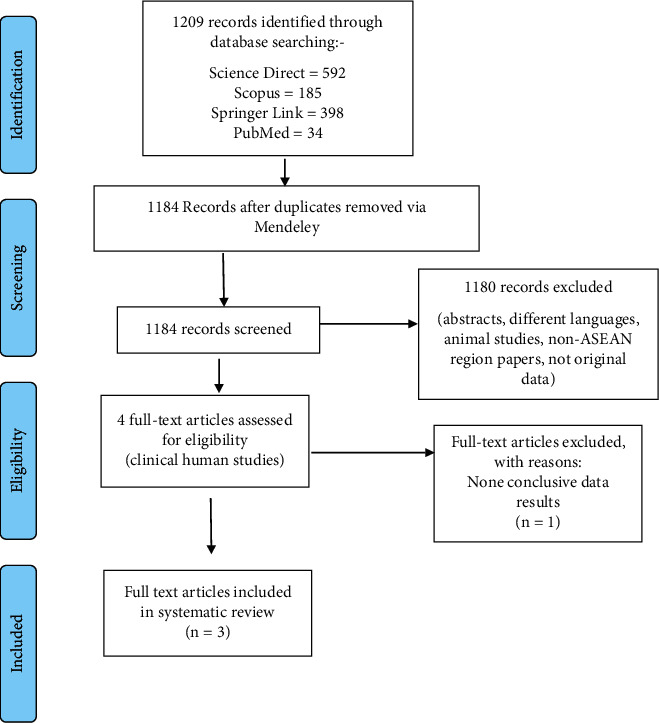
PRISMA flow diagram of study selection of initial and updated literature searches (inclusion and exclusion) for the systematic review.

Using the PRISMA diagram as guidance (Figure 1) which includes identifying and screening of articles from databases, relevant articles were selected based on identified medicinal plants believed to be antidiabetic in their respective countries in which these plants have been used as an intervention on prediabetic or type 2 diabetic participants in ASEAN countries and assessed outcome measures related to the management of type 2 DM such as lowering of blood glucose amongst participants upon completion of the intervention in varying timeframes.

Two independent reviewers evaluated all reference titles obtained from all selected databases. Then, abstracts were used as guidance by the two reviewers to assess and determine whether the articles met the selection criteria for the full review. Any discrepancies with the selected articles were settled by having a third independent reviewer reach a consensus.

### 2.3. Data Extraction

Data extracted from each study include title, author(s), journal, year and country of publication, study characteristics (study design, study duration), characteristics of participants (gender, age, sample size), plant type and extractions, intervention (type of treatment) and control used, number of dropouts, methods, and results from the studies. These studies are summarized in Table 1.

### 2.4. Methodological Quality Assessment

Final identified studies were assigned to three reviewers, whereby two reviewers have independently completed data extraction and assessed the quality of studies using the Downs and Black quality assessment tool, which is a well-used and validated numerical rating scale for assessment of the methodological quality of both randomized and nonrandomized studies [15]. The 27 criteria of the Downs and Black assessment tool can be used for both randomized and nonrandomized studies and assesses study reporting (10 items), external validity, that is, the representativeness of findings based on the study population (3 items), and internal validity (14 items) which include bias and power, with a maximum scoring of 32 points.

## 3. Results

A total of 1209 reference titles were identified from the literature search of the four mentioned selected databases. Using Mendeley as the database manager, these underwent further screening to remove any duplicates (*n* = 25). Two independent reviewers completed further screening, to remove nonrelevant articles that do not have full-text articles, articles of non-English language, and studies without human trials, including only ASEAN region papers and original data.

From the screening, only four articles were selected for full reviews; however, one article was eliminated as the study did not provide a conclusive outcome with measured data (Figure 1), of which, out of the three selected studies, two studies were randomized clinical trials.

### 3.1. Comparison of Methodological Quality Using Downs and Black Checklist

The three selected articles for full review underwent quality assessment using the Downs and Black tool, whereby, overall, the mean score was 18, with a grade as being “fair” (14–18 points). One article scored the highest (22 points) and was graded as “good” (19–23 points), providing detailed study design and clear external and internal validities, while two other articles scored the same (16 points) and were graded as “fair” (14–18 points) (15).

### 3.2. Countries Included and Study Outcome

After the rigorous screening of literature searches for human clinical studies in ASEAN countries, only two Southeast Asian countries were included for review: the Philippines (1 study) and Indonesia (2 studies). Only the nonrandomized (quasi-experimental) study used a chosen medicinal plant, rosella flower (*H. sabdariffa* L.) in its natural form, as ready-to-drink tea with an added natural sweetener, stevia. On the contrary, the two randomized-controlled studies used their selected medicinal plants in capsule forms, that is, existing commercial products acting as supplements, bitter melon (*M. charantia*), and combination extracts as *Allium Curcuma* of turmeric (*C. longa*) and garlic (*A. sativum* L.) in The Philippines and Indonesia, respectively.

Only the 3-month bitter melon (capsule form) study as opposed to the 14-day to 12-week studies in Indonesia on adult diabetic participants showed no significant difference in their fasting blood glucose, as well as their cholesterol level. However, consumption of rosella brewed in tea form for 14 days, as well as drug *Allium Curcuma* for 12 weeks, showed a significant difference in participants' fasting blood glucose for both studies, as well as 2 hours postprandially, and HbA1c level for the latter intervention.

### 3.3. Medicinal Plants

#### 3.3.1. *Curcuma longa* (Turmeric) with *Allium sativum L*. (Garlic)

Sukandar et al.'s study [16] consisted of 35 diabetic Indonesians and their responses to the study drug, *Allium Curcuma* capsule (200 mg turmeric and 200 mg garlic) against a standard drug, 5 mg of glibenclamide, of which the former resulted in a significant decrease in fasting and the 2-hour postprandial blood glucose. There were no changes to the participants' blood pressure, hematology profile, and liver and kidney function and no harmful drug interactions during the study.

#### 3.3.2. *Hibiscus sabdariffa* L. (Roselle Plant)

The quasi-experimental research study conducted by Mayasari et al. [17] was a 14-day treatment of prediabetic women (aged 30–60 years), using ready-to-brew rosella powder (5 g) with 125 mg stevia, and showed a significant reduction of fasting blood glucose of treatment group compared to the control group. However, there was no significant difference observed at 2-hour postprandial blood glucose results between the control and treatment group after the two-week treatment.

#### 3.3.3. *Momordica charantia* (Bitter Melon)

Dans et al. [18] conducted a randomized, double-blind, placebo-controlled trial testing the efficacy of *M. charantia* (bitter melon) on the control of diabetes. The primary outcome of the study was the change in HbA1c level after treatment. Unlike previous nonrandomized trials on the plant, the authors of this study concluded that consumption of *M. charantia* had no effect on the HbA1c levels of the diabetic patients enrolled in the study.

## 4. Discussion

From ancient times to the present, medicinal plants continue to play an important role in providing therapeutic assistance for various ailments and in this case for the treatment of diabetes for both developing and developed countries around the world. According to the WHO, 21 000 plants around the world have been listed to provide medicinal values. Over one billion people still rely heavily on the healing properties of medicinal plants, and at least 150 plants have already been used for pharmaceutical purposes on a large scale [19].

To ensure the protection of medicinal plants from uncontrolled harvesting from their natural habitat, the WHO [20] has developed the *Guidelines on Good Agricultural and Collection Practices (GACP) for Medicinal Plants*. This guide aims to advise users of medicinal plants on how to practice sustainable harvesting of these plants, as well as how to carry out strategic consumption of the finished herbal products to reduce unnecessary wastage.

Hence, medicinal plant products in capsule, tea, or powdered form are more readily available commercially and easily accessible to the public for consumption as opposed to the olden days.

### 4.1. Turmeric (*C. longa*) with Garlic (*A. sativum* L.) and Diabetes

**Figure 2:**
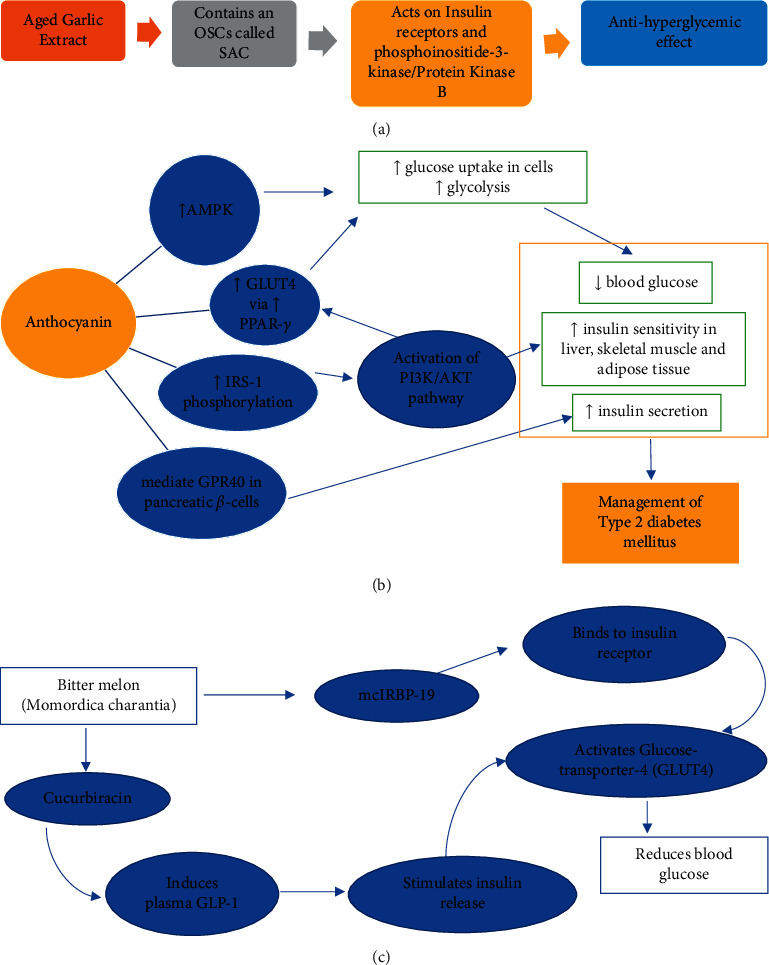
. Possible mechanisms of bioactive compounds. (a) Proposed mechanism in garlic, (b) proposed mechanism of anthocyanin in rosella plant, and (c) proposed mechanism in bitter melon to manage hyperglycaemia.

The well-known pungent smell of garlic is attributed to the presence of organosulfur compounds (OSCs), which are also antioxidants, antihypertensive, and antidiabetic [21, 22]. Allicin, one of the major OSCs from crushed garlic, is converted from alliin (S-allyl-cysteine sulfoxide) producing other forms of OSCs via enzymatic reactions, such as diallyl monosulfide (DAS) and oil-soluble polysulfides, diallyl disulfide (DADS), and diallyl trisulfide (DATS) [21]. These rapid reactions resulted in the abundance of OSCs for good glycemic control [22], as suggested in Figure 2(a) using aged garlic extract (adapted from Melino et al. [21]).

For almost 80 years, studies on garlic on blood glucose control have already existed. Various types of garlic preparations (such as ethanol, 40/60 petroleum ether, and diethyl ether extracts of garlic) have been tested in humans and animal studies [23]. One of the earlier studies by Jain and Vyas [24] reported a good effectiveness range of 61–93% of the various garlic extracts compared to the efficacy of a single dose of tolbutamide in alloxan diabetic rabbits. Some examples of garlic preparations include aged garlic extract, fresh garlic in tablet form, raw garlic, fresh garlic, and fermented garlic extract by *Bacillus subtilis*, 1.5% black garlic extract [22].

Further investigation has shown these studies favor the use of fresh garlic extraction for both animal and clinical works to measure antidiabetic properties in garlic. Wang et al. [25] reported garlic reduced fructosamine and glycosylated hemoglobin in clinical trials and was shown to provide a protective effect against diabetic retinopathy and improved blood glucose and morphological changes of retinal tissue in rats [26].

### 4.2. Turmeric and Diabetes

Turmeric, the root of *Curcuma longa* L., originates from a member of the ginger family [27], readily available in powdered form as cooking spices, food additives, and cosmetics, as well as in pharmaceutical industries [28].

Similar to garlic, the uses and health benefits of turmeric are well-researched, especially on curcuminoids, an important bioactive compound [29] that acts as a potent antioxidant, has anti-inflammatory effects, is hepatoprotective, and has antidiabetic properties [30–32]. There are three types of chemically related components of curcuminoids: curcumin (C), demethoxycurcumin (DMC), and bis-demethoxycurcumin (BDMC) [33]. Besides curcuminoids, ar-turmerone and tumerin are other types of bioactive compounds found in turmeric, which are also believed to be antidiabetic [31].

Hartogh et al. [30] recently reported that curcumin was able to improve beta-cell function, thus reducing diabetic progression amongst prediabetic and diabetic patients, whereby oral consumption of curcumin was found to be safe when taken at a dose of 6 g/day for 4–7 weeks [34]. Soleimani et al. [34] further reported that oral intake of curcumin is safe for human consumption at a dose of 500 mg twice a day for 30 days. However, a recent animal study has shown that there was a possible potential risk of liver damage derived from high oral administration of curcumin at 250 mg/kg twice weekly [35]. Oral administration of turmeric extract and curcumin, however, showed protective effects on the pancreatic and renal functions of diabetic rats [35, 36]. Therefore, more studies are required to look into the safe and effective consumption of curcumin [30].

Apart from lowering the blood glucose levels, the intake of turmeric also reduces complications resulting from diabetes such as cardiovascular disease and retinal neuropathy. Srinivasan et al. conducted a three-month randomized-controlled trial with *C. longa* L. which reported arterial stiffness was reduced significantly as compared with placebo in type 2 diabetes mellitus patients [37]. Banafshe et al.'s study [38] on diabetic rats has shown turmeric administration resulted in a protective effect against retinal neuropathy, overall demonstrating the protective effects of turmeric for various internal body systems.

### 4.3. *Hibiscus sabdariffa* L. and Diabetes

A woody-based subshrub, *H. sabdariffa* L. (roselle plant), belongs to the Malvaceae family, with more than 300 species known to be widely cultivated in both the tropical and subtropical regions of the world [39]. Traditionally and up to the present, the *H. sabdariffa* L. calyces have been used for various purposes in the food industries as well as medicinal purposes, in the form of herbal drinks and flavoring agents and as herbal medicine [40, 41] due to its bright red color and sour flavoring, which is mainly attributed to the high presence of anthocyanins.

Anthocyanins, a subgroup of flavonoids, have been reported to give hypoglycemic effects [42] in several studies. Sancho et al. [43] reported several experiments *in vitro* and *in vivo* have already been conducted to prove the antidiabetic properties of anthocyanins, including increased insulin secretion, improved insulin resistance, and lowered blood glucose, as also shown in several animal studies [44–46] using different breeds of rats and duration of intervention.

Mayasari et al.'s study [17] showed that a 14-day intervention using rosella-stevia tea resulted in a significant reduction in the fasting blood glucose of prediabetic women, attributed by the active compounds present, which may be due to not only the high antioxidant properties of *H. sabdariffa* L. but potentially also enzymatic inhibition and other pathways.

Using the High-Performance Liquid Chromatography (HPLC) and Liquid Chromatography-Mass Spectrometry (LC-MS) [47], comparisons were made using various solvents to identify the maximum extraction of the bioactive compounds, which are predominantly delphinidin, cyanidin 3-sambubioside, and cyanidin 3-glucoside [41]. The highest amount of anthocyanins was extracted in water as opposed to using solvents such as methanol, ethyl acetate, and hexane in the presence/absence of formic acid, with different extraction times and temperatures [47]. Hence, the study concluded that the maximum extractable compounds and yields of anthocyanins are in an aqueous solution, for 10 minutes at 100 degrees Celsius. These results were further explained through the nature of the identified anthocyanins' structures, that is, having high hydrogen-donating capacity; hence, maximum extractions are highly dependent on the polarity of the solvent, with water being the highest compared to the rest of the mentioned solvents [48–50].

Therefore, Mayasari et al.'s positive results [17] can be justified due to the scientific evidence presented in *in vitro* and *in vivo* studies, whereby participants were given strict instructions on the correct tea preparation method before consumption, that is, 5 g of rosella powder with 125 g of stevia in boiling water for 5 minutes.

Although the mechanisms and pathways of anthocyanins of *H. sabdariffa* L. are not completely clear, Peng et al. [45] attributed the reduction in blood glucose and improved insulin sensitivity to the mediation of GPR40 in pancreatic beta-cells and the insulin receptor substrate-1 serine kinase that regulates the insulin receptor signal pathway. The improvement was shown in the number of pancreatic *β* cells and reduction in blood glucose level in streptozotocin-induced diabetic rats [51]; it also showed no correlation was observed between the level of serum insulin and lowering of blood glucose, indicating presence of insulin resistance in type 2 diabetic rats group. One study using mulberry anthocyanin extract reported reduced fasting blood glucose and serum insulin in db/db mice, whereby *in vitro* further investigation showed the activation of PI3K/AKT pathways in HepG2 cells that improves insulin resistance and increased glucose consumption, glucose uptake, and glycogen content [52].

Hence, the possible mechanism of anthocyanins on glucose metabolism is proposed to affect insulin secretion and improve insulin sensitivity and glucose uptake in the liver, adipose tissues, and skeletal muscles (Figure 2(b)) to lower blood glucose, via GPR40, AMPK, GLUT4 AMD PI3K/AKT pathways.

Further investigation needs to be done to understand the mechanisms associated with improved insulin production, sensitivity, and reduced serum blood glucose.

### 4.4. *Momordica charantia* and Diabetes

*M. charantia* is one of the most frequently ingested herbs for the control of diabetes in complementary and alternative medicine users in Malaysia and India, as well as users of traditional Chinese medicine [53–55]. Bioactive compounds that have been identified within the plant include charantin, an insulin-like peptide, cucurbitane glycosides, momordicin, and oleanolic acids [56, 57]. Both animal and human studies have suggested that the active compounds within the plant can exert hypoglycemic effects [58, 59]. However, no hypoglycemic effect was identified by Dans et al. [18], which was the only study available on *M. charantia* found in this systematic review.

Both Dans et al. [18] and Cortez-Navarette et al. [58] carried out randomized-controlled trials (RCTs). However, different bioactive compounds have been identified in *M. charantia* extracts collected from different regions in China [55]. The differing results obtained by the two RCTs may therefore be due to the different compounds available in the *M. charantia* plants in the Philippines and Mexico.

Despite the negative results reported by Dans et al. [18], they suggested a study with a bigger test population may be able to detect more modest effects on blood glucose. It has recently been reported that lactic acid fermented *M. charantia* juice lowered fasting and postprandial blood glucose levels in rats more efficiently than nonfermented *M. charantia* juice [60]. Therefore, it may be useful to use a similar fermented juice in human trials as well, in order to accentuate the antidiabetic effects of the plant which may have been masked in the Dans study [18]. Another avenue of research that seems promising is the use of a bitter melon peptide mcIRBP-19 in controlling blood glucose levels in diabetic patients, as this may give a more concentrated dose of antidiabetic bioactive compounds to the patients [61]. However, it should be noted that *M. charantia* was shown to have developmental toxicity on zebrafish embryos, so it should be taken with caution, particularly by those who are or planning to be pregnant [62].

Figure 2(c) illustrates possible mechanisms involved in *M. charantia* of cucurbitacin and mcIRBP-19 which activates GLUT4 to increase glucose uptake into cells to lower blood glucose and assist in the management of diabetes.

## 5. Conclusion

This review demonstrates the limitations in published articles on human clinical trials for medicinal plants' intervention for diabetes. Upon further investigations on the four identified medicinal plants that were mostly done in animal studies, findings showed positive effects in the management of diabetes, such as hyperglycemia. The varying methods and extractions used on different medicinal plants in different parts of the world are also worth noting, which is likely attributed to the inconsistencies in data results. The most used and reliable extractions of medicinal plants are the powdered form, which are then to be diluted in either aqueous or alcoholic solutions prior to intervention, especially in animal studies. Hence, further testing and standardization of the methods in the studies can be suggested for human clinical trials for reliable data collections such as methods of extract preparation, duration of intervention, and conditions set for the study design.

## Figures and Tables

**Table 1:** Completed summary of all data extractions of selected published literature articles on human studies and medicinal plants in the management of type 2 diabetes mellitus in ASEAN countries.

Author(s), year of publication, study design	Plant name and type of extraction	Country participant description, no. of dropouts	Duration and type of treatment	Study outcome	Brief methods, e.g., blood test
Mayasari et al., 2018	Rosella with stevia	Yogyakarta, Indonesia	14-day treatment	Posttreatment	Venous blood samples collected for FBG and 2-hour PBG
Quasi-experimental research study	As ready-to-brew rosella-stevia bags	24 prediabetic women	(1) Fast for 8 hours the night before the first day of treatment, to withdraw participants' blood for fasting blood glucose readings	Tea consumption significantly lower FBG level but not the 2-hour PBG level	
	(i) 5 g rosella powder				
	(ii) 125 mg stevia sweetener	Aged 30–60 years			
		2 groups (control and treatment group)	(2) Each participant was given 75 g sugar in 250 ml water and rested for 2 hours, for 2^nd^ blood withdrawal as 2-hour postprandial blood glucose		
	Each rosella-stevia tea bag is brewed with 250 ml of boiling water for 5 mins and cooled down for 20–30 minutes before consuming	3 dropouts (80% less compliance)	(3) Treatment group		
			(i) Ready-to-brew rosella-stevia tea 2x per day for 14 days		

Dans et al., 2007	*M. charantia (Bitter melon)*	Manila, Philippines	3-month treatment:	No significant effect on mean FBG, total cholesterol, and weight or on serum creatinine, ALT, AST, sodium, and potassium	Each monthly visit:
Randomized double-blind, placebo-controlled trial	As *charantia ampalaya* capsules vs. placebo capsules	40 patients with newly diagnosed or poorly controlled type 2 diabetes with A1c levels between 7% and 9%	(1) *M. charantia* capsules or placebo:		(i) Capillary blood sugar levels
			(i) 2 capsules, 3 times per day after meals for 3 months		(ii) Interviewed on compliance and adverse events
		Aged 18 years and above	(ii) Monthly follow-ups		(iii) Diet and medications reinforced
					Lab test:
					(i) HbA1C
					(ii) Fasting blood glucose
					(iii) Serum cholesterol
					(iv) Weight
					Extra:
					(i) Serum creatinine, AST, ALT, sodium, potassium
					Adverse events

Sukandar et al., 2014	*Curcuma longa* (turmeric) with *A. sativum* L. (garlic)	Bandung, Indonesia	For 12-week treatment:	(i) Significant decrease in FBG of AC group (192.76 vs. 141.71 mg/dL) and 2 hours postprandial blood glucose (295.35 vs. 204.35 mg/dL)	Evaluated every 2 weeks for 12 weeks
Double-blind, randomized control trial	As drug (allium curcuma) in the form of capsules:	36 patients with type 2 diabetes (males and females) aged over 35 years	(1) AC group:		(i) Fasting blood glucose
	(i) 200 mg of turmeric ethanolic extract, 200 mg of garlic aqueous extract	(i) Random blood glucose at least 200 mg/dL, fasting blood glucose level at least 126 mg/dL with or without dyslipidemia	(i) 2.4 g *Allium curcuma* capsules		(Ii) 2 hours postprandial
	Vs.		(ii) 2 times, 3 capsules per day after meal		(iii) Lipid profile examination
	Oral drug		(2) Glibenclamide group:	(ii) Significant decrease in HbAlC 10.41 vs. 8.09)	On week 2 and week 14, examination on:
	(ii) 5 mg glibenclamide	2 groups	(iii) 2 times 3 capsules per day		(iv) HbA1C. Fasting insulin, liver function, renal function, complete hematology, urine and heart function
		AC group = 17	(iv) But for morning after meal, each consumed 1 capsule of 5 mg glibenclamide, and 2 capsules as placebo		
		Glibenclamide group = 12	(v) Evening after meal, each consumed 2 placebo capsules		

Data extracted from each study include title, author(s), journal, year and country of publication, study characteristics (study design, study duration), characteristics of participants (gender, age, sample size), plant type and extractions, intervention (type of treatment) and control used, number of dropouts, methods, and results from the studies.

## Data Availability

No data were used to support the findings of this study.
